# The genomic distribution of transposable elements is driven by spatially variable purifying selection

**DOI:** 10.1093/nar/gkad635

**Published:** 2023-08-10

**Authors:** Anna M Langmüller, Viola Nolte, Marlies Dolezal, Christian Schlötterer

**Affiliations:** Institut für Populationsgenetik, Vetmeduni Vienna, Veterinärplatz 1, 1210 Wien, Austria; Vienna Graduate School of Population Genetics, Vetmeduni Vienna, Veterinärplatz 1, 1210 Vienna, Austria; Institut für Populationsgenetik, Vetmeduni Vienna, Veterinärplatz 1, 1210 Wien, Austria; Plattform Bioinformatik und Biostatistik, Vetmeduni Vienna, Veterinärplatz 1, 1210 Vienna, Austria; Institut für Populationsgenetik, Vetmeduni Vienna, Veterinärplatz 1, 1210 Wien, Austria

## Abstract

It is widely accepted that the genomic distribution of transposable elements (TEs) mainly reflects the outcome of purifying selection and insertion bias (1). Nevertheless, the relative importance of these two evolutionary forces could not be tested thoroughly. Here, we introduce an experimental system, which allows separating purifying selection from TE insertion bias. We used experimental evolution to study the TE insertion patterns in *Drosophila simulans* founder populations harboring 1040 insertions of an active P-element. After 10 generations at a large population size, we detected strong selection against P-element insertions. The exception were P-element insertions in genomic regions for which a strong insertion bias has been proposed (2–4). Because recurrent P-element insertions cannot explain this pattern, we conclude that purifying selection, with variable strength along the chromosomes, is the major determinant of the genomic distribution of P-elements. Genomic regions with relaxed purifying selection against P-element insertions exhibit normal levels of purifying selection against base substitutions. This suggests that different types of purifying selection operate on base substitutions and P-element insertions. Our results highlight the power of experimental evolution to understand basic evolutionary processes, which are difficult to infer from patterns of natural variation alone.

## Graphical Abstract

**Graphical Abstract:**
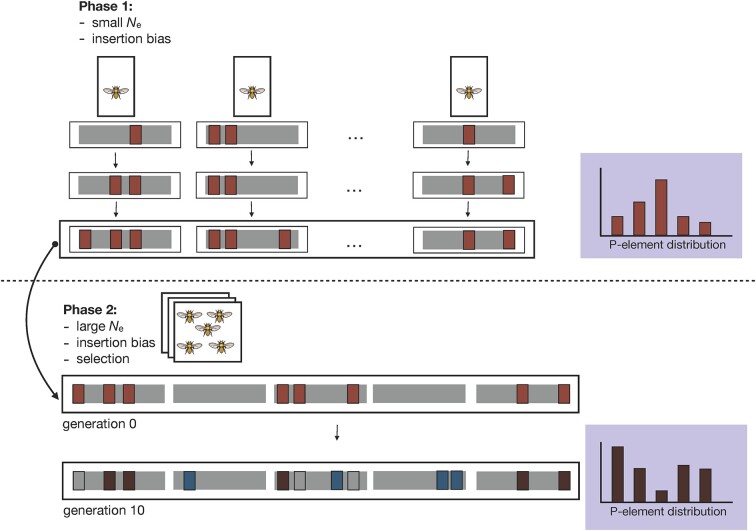

## INTRODUCTION

Transposable elements (TEs) are DNA sequences that move and amplify within the host genome (5). Their diverse structures and proliferation mechanisms (1) are well-documented across the entire tree of life (1,6). The dynamics of TEs are not only driven by their selfish amplification mechanisms, but also by the fitness consequences for the host mediated by TE insertions. This interesting interplay has attracted the attention of evolutionary biologists since the discovery of TEs and continues to do so.

Purifying selection is one of the key evolutionary forces shaping the distribution of TEs in natural populations (1,6) and three different mechanisms of TE-related fitness costs have been discussed (7). (i) RNAs or proteins required for transposition are deleterious to the host (6,8), (ii) TE copies at non-homologous sites cause elevated ectopic recombination rates (9–11), (iii) new TE insertions are deleterious for the host (7,12–14).

Empirical evidence for purifying selection removing TE insertions is either based on ‘patterns of absence’ in functionally important regions of the host genome (e.g. exons) (15,16) or low population frequencies in natural populations (17). This inference makes the implicit assumption of a similar insertion rate of TEs across the entire genome. Since insertion preferences of TEs have been reported (1,3,18) the distinction between purifying selection purging deleterious TE insertions and insertion bias is difficult, if not impossible, when only the genomic distribution of TEs of one time point is used to infer evolutionary TE dynamics. Thus, it has been suggested to study TE dynamics under controlled environmental conditions and varying selection pressures (19,20), but such studies are rarely carried out.

**Figure 1.**
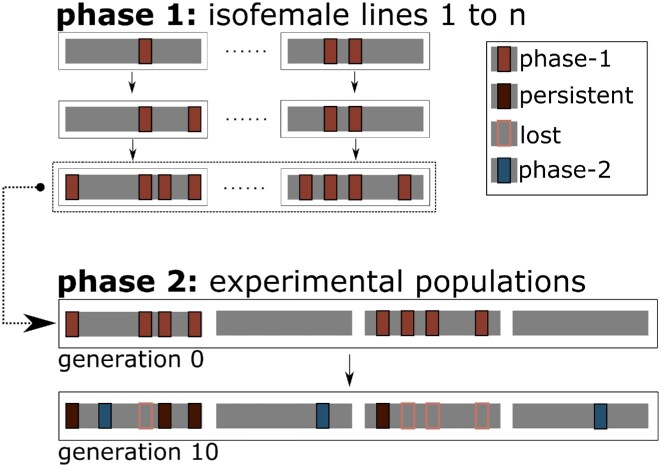
Schematic overview of the experimental design. Phase 1 (low efficacy of purifying selection due to small population sizes): acquisition of P-element insertions (red rectangles) during 4.5 years in small populations (isofemale lines with ∼40–50 individuals). Phase 2 (high efficacy of purifying selection due to large population sizes): measuring purifying selection for two classes of P-element insertions: persisting insertions from phase 1 (dark red) and insertions acquired during phase 2 (blue). Isofemale lines with and without P-elements were combined to form three large outbred replicate populations in phase 2 (*N* = 1250 individuals per replicate).

In this study, we use Evolve & Resequence (E&R) (21–23) to distinguish the relative importance of insertion bias and purifying selection on the genomic distribution of TE insertions. We take advantage of a natural *D. simulans* population that is invaded by the P-element (4). The P-element—a 2.9 kb long DNA transposon (24,25)—is one of the best studied TEs in eukaryotes (26) and the successful invasion of different *Drosophila* species is well-documented (4,27–29). To distinguish insertion bias from selection, our study consisted of two phases, which differed by the opportunity for purifying selection: In phase 1, the P-element proliferated in small populations (isofemale lines) with an active P-element (29,30) for about 4.5 years. In phase 2, we established large outbred populations by mixing isofemale lines with and without P-elements and evolved them for 10 generations. This experimental design allowed us to disentangle purifying selection from insertion bias (Figure 1). While purifying selection is particularly strong in exons, we also found negative selection against P-element insertions in introns and intergenic regions. Very weak selection occurred against P-element insertion sites that have been previously reported to be shared between natural *Drosophila melanogaster* and *D. simulans* populations (4).

## MATERIALS AND METHODS

### Set up and maintenance of experimental populations

We set up three independently maintained replicate populations in June 2015 using 191 isofemale lines collected from a natural *D. simulans* population in Tallahassee, Florida (31). It has been estimated that 25–44% of these isofemale lines contain at least one copy of the P-element (4,29). At the time of set up, the isofemale lines had been maintained for 4.5 years at small population sizes (∼40–50 individuals). Each replicate population consisted of five 300 ml plastic bottles with 70 ml standard *Drosophila* medium, and each of these bottles was set up with 191 mated female flies – one female from each of the isofemale lines (corresponding to the F0 flies). The offspring of all five bottles in a given replicate were mixed before the set-up of each new generation. Replicates were maintained with non-overlapping generations and a census size of 1250 flies in a cycling hot temperature regime (12 h light and 28°C; 12 h dark and 18°C).

### P-element detection

#### Isofemale lines

To test whether P-element abundance has increased in isofemale lines over time (Supplementary Figure S1, Supplementary File 1), we estimated P-element copy numbers in three different isofemale lines (I116, I174, I211) after 2 and 6 years of maintenance in the laboratory. Isofemale lines were collected from a natural *Drosophila* population in Tallahassee Florida in November 2010 (31) and have been kept at small population sizes (∼40–50 individuals) ever since. The details of the genomic sequencing and crossing schemes can be found in (4) and (32). For each time point, one individual male from an isofemale line was crossed with a virgin female from the P-element free M252 reference strain (4,33). From each cross, one single female offspring was sequenced. We demultiplexed barcoded files (*–maximumMismatches 3 –maximumN 2*) and trimmed raw paired-end reads (*–mottQualityThreshold 15 –disable5pTrim –minReadLength 25*) with ReadTools (v1.5.2) (34). We estimated P-element copy numbers with DeviaTE (35). Because DeviaTE does not take paired-end read information into account (35), we only used the first read from each read pair for the analysis. To avoid systematic biases in P-element copy number estimates due to different read lengths (35) (2 years: 100 bp, 6 years: 150 bp), we clipped the quality-trimmed reads to a maximum read length of 100 bp using the program BBDuk of BBMap (v38.87; sourceforge.net/projects/bbmap). We mapped the resulting trimmed reads with bwa bwasw (v0.7.17; *–M*) (36) to a reference containing the P-element consensus sequence of *Drosophila* (37) and three single-copy genes (*rpl32, traffic jam, rhino*). DeviaTE estimates P-element copy numbers per haplotype by comparing the read depth of single copy genes with the read depth of the P-element consensus sequence (35,38).

#### Experimental populations (phase 2)

We sequenced the ancestral populations (*n* = 3, generation 0, females only) and the evolved populations (*n* = 3, generation 10, mixed sexes) using the Pool-Seq (39) approach. Genomic DNA was extracted with a standard salting out protocol (40) from the pool of all females for a given replicate in the ancestral population (5 × 191 flies = 955 flies) and for half of the pooled individuals from a given replicate in the F10 generation (approx. 600 flies). For the ancestral populations, paired-end libraries were generated using the NEBNext Ultra II DNA Library Prep Kit, starting from 1000 ng DNA sheared with a Covaris S2 (Covaris, Inc. Woburn, MA, USA). Libraries were subjected to double-sided size selection for an insert size of 300 bp using AMPure XP beads (Beckman Coulter, Carlsbad, CA) and amplified with 4 PCR cycles using dual index primers. For generation 10, paired-end libraries were generated using the NEBNext Ultra II FS DNA Library Prep Kit (New England Biolabs, Ipswich, MA). The protocol was modified to use only 10% of the provided reagents in each step, a target insert size of 300 bp and amplification with dual-index primers and 5 PCR cycles. For the experimental populations in phase 2, all libraries were sequenced on a HiSeq X Ten with a read length of 2 × 150 bp.

We used ReadTools (v1.5.2) (34) to demultiplex barcoded paired-end read data (*–maximumMismatches 3 –maximumN 2*) and trim reads (*–minReadLength 25 –mottQualityThreshold 15 –disable5pTrim*). To increase physical coverage (i.e. the number of paired reads spanning a genomic site, where only the inner distance is considered) (41), we clipped the quality-trimmed reads to a maximum read length of 75 bp using the program BBDuk of BBMap (v38.87; sourceforge.net/projects/bbmap). As recommended (41), we mapped reads in single-end mode using bwa bwasw (v0.7.17; *–M*) (36) to a reference containing the P-element free M252 reference genome (4,33) and the consensus sequence of the *Drosophila* P-element (37). We restored paired-end information with PopoolationTE2 (v1.10.03, *se2pe*) afterwards (41).

#### P-element classification (joint analysis)

For each sample (*n* = 6; three replicate populations sequenced at generation 0 and 10), we used samtools (v1.11; *merge*) (42) to merge all available reads before we generated a ppileup file with PopoolationTE2 (v1.10.03; *ppileup*) (41) that contained all six samples. To avoid differences in sensitivity due to coverage heterogeneity, we subsampled the resulting ppileup to a uniform physical coverage of 50x per sample (41), which removed 14.96% of the genomic sites from the analysis. P-element signatures were identified on the main chromosome arms (X, 2L, 2R, 3L, 3R, 4) with the joint analysis mode of PopoolationTE2 (v1.10.03; *identifySignatures –signature-window minimumSampleMedian –min-valley minimumSampleMedian –min-count 1*) (41). PopoolationTE2 uses a sliding window-based approach to detect peaks in the physical coverage that support a P-element insertion. For the final set of P-element insertion sites, these signatures are then merged if they are within a given distance (500 bp). We masked P-element signatures in low complexity regions (<= 200 bp; identified by RepeatMasker (http://www.repeatmasker.org, v4.0.7), in previously reported Y-translocations (43), and in regions with an average coverage <50× (PopoolationTE2 v1.10.03 *filterSignatures –min-coverage 50*) before calling final P-element insertions with PopoolationTE2 (v1.10.03 *pairupSignatures*, Supplementary File 2) (41).

For each replicate population, P-element insertions are classified as ‘lost’, if they are detected in any replicate population after phase 1 (i.e. generation 0), but are not detected in the focal replicate population after phase 2 (i.e. generation 10), whereas ‘persistent’ P-element insertions are detectable after phase 2. P-element insertions, which occurred during phase 2 in the focal replicate population, and are not detectable after phase 1 in any replicate population, are classified as ‘phase-2″ (i.e. new insertions). Given that most P-element insertions occur at a low frequency, not all insertions can be re-discovered, which could lead in a false classification of P-element insertions. Nevertheless, given that the frequency of P-element insertions is very similar across genomic annotation features (Supplementary Figure S2A), we expect the same re-discovery and false classification rate for all genomic annotation features.

#### P-element frequency distribution

The joint analysis mode of PopoolationTE2 is well-suited to study orthologous P-element insertion sites in different samples, but it is not well-suited for allele frequency estimates across multiple evolutionary replicates: low frequency P-element insertions of the same replicate population sequenced in a time-resolved manner are more likely to be detected and included in the frequency estimates (41). To estimate the frequency distribution of P-element insertions, we generated a ppileup file (PopoolationTE2; v1.10.03; *ppileup* (41)) that contains only the three replicate populations sequenced at generation 0. PopoolationTE2 estimates population frequencies of TEs as the proportion of physical coverage supporting a TE insertion to the total physical coverage of the population. To obtain roughly the same sensitivity as in the joint analysis with six samples, we subsampled the resulting ppileup to a uniform physical coverage of 60x per sample, which removed 19.12% of the genomic sites from the analysis. We identified and filtered P-element insertions as described above (with the exception of masking regions with an average coverage <60× instead of <50×), before estimating frequencies of individual P-element insertion sites with PopoolationTE2 (v1.10.03 *pairupSignatures*, Supplementary File 3) (41).

#### P-element classification (separate analysis)

To confirm the robustness of our results, we reanalyzed the P-element insertions for each replicate population independently. For this, we generated for each replicate population (*n* = 3) an individual ppileup file with PopoolationTE2 (v1.10.03; *ppileup*) (41) that contained the respective experimental population sequenced at generation 0 and 10. To obtain roughly the same sensitivity as in the joint analysis with six samples, all resulting ppileup files were subsampled to a uniform physical coverage of 60x per sample (41), which removed 15.61%, 19.13% and 16.27% of the genomic sites from the replicate-specific analysis. P-element signatures were identified as described above (with the exception of masking regions with an average coverage <60× instead of <50×) before calling final P-element insertions with PopoolationTE2 (v1.10.03 *pairupSignatures*, Supplementary File 4) (41).

In a replicate population, a P-element insertion is classified as ‘lost’, if it is present after phase 1 (i.e. generation 0), but is not detected after phase 2 (i.e. generation 10). ‘Persistent’ P-element insertions are detectable after both phase 1 and phase 2, and ‘phase-2' P-element are only detectable after phase 2. In the ‘separate analysis’, P-element insertions are considered to be independent across replicate populations (i.e. no identification of orthologous insertion sites). The only exception is the replicate frequency spectrum: here, P-element insertions with a distance of less than 1 kb across replicate populations are considered to be identical. Identical P-element insertions were determined with bedtools merge (v2.29.2) (44).

We observed that the joint and separate analyses resulted in qualitatively similar results. Thus, we present the joint analysis in the main part of this manuscript and show the results of the separate analysis in the Supplement.

#### Natural population

To compare the genomic distribution of P-element insertions of our experimental populations to a natural population, we annotated previously reported P-element insertion sites from a wild *D. simulans* population from South Africa with an established P-element invasion (4). For this, we obtained P-element insertion sites together with accompanying annotations and reference genomes from the original analysis by Kofler *et al.* (4,33).

### Characterization of P-element insertion sites

#### Recombination rate

We used the *D. simulans* recombination map from Howie *et al.* (file: Dsim_recombination_map_LOESS_100kb_1.txt) (32) to check the average recombination rate in cM/Mb for each P-element insertion site in experimental populations. We used the 10th percentile (= 2.85 cM/Mb) of the genome-wide recombination rate as a threshold determining whether P-element insertions are in a region with low recombination rate.

#### Annotations

##### Genomic features

For both, experimental and natural populations, we used bedtools sort, merge, intersect and complement (v2.29.2) (44) to extract different features from the *D. simulans* annotation (4,33) in order to determine the annotation type of each P-element insertion. In case different annotation tracks were overlapping, we recorded the most detailed annotation (e.g. coding sequence over translated region) and the annotation with the presumably higher functional importance (e.g. exon over intron).

For each population, we calculated the expected number of P-element (*P_exp_*) insertions in a respective genomic feature as follows:


(1) \begin{equation*}{P}_{exp} = {\mathrm{\ }}\frac{{{f}_l{\mathrm{*}}{P}_{obs - total}}}{{{G}_l}}\end{equation*}

**Figure 2.**
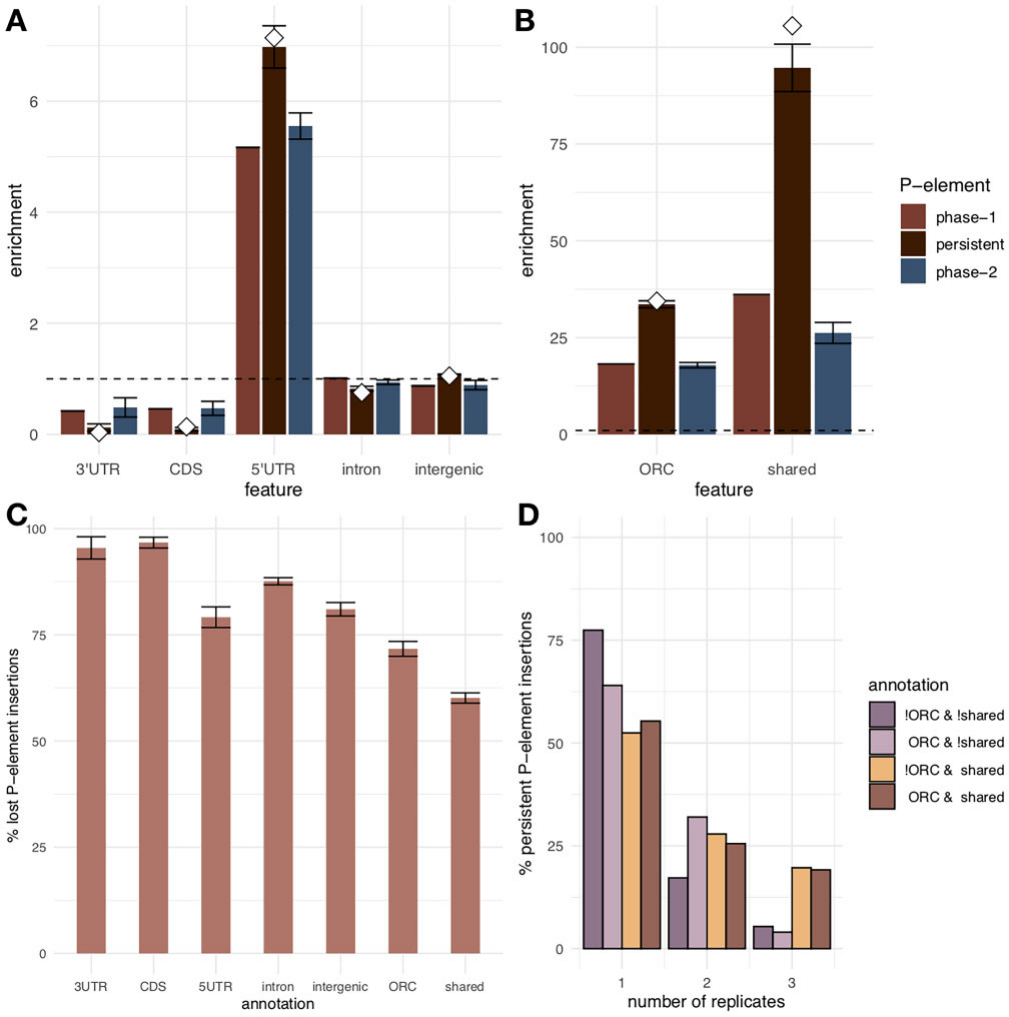
Characteristics of P-elements in phase 1 and phase 2. (**A**) Average enrichment (=observed/expected) of different P-element classes by annotation feature across three replicate populations. The horizontal dashed black line marks a homogeneous distribution (i.e. no insertion bias or selection, observed = expected). Nevertheless, since we consider the enrichment in the 5′UTR to reflect reduced purifying selection in combination with insertion bias, the lower enrichment values of all other functional classes indicate purifying selection. White diamonds display the enrichment of P-elements across genomic features for a natural *D. simulans* population from South Africa with an established P-element invasion (4).The error bars show the standard error of the mean across experimental populations. (**B**) Average enrichment of all phase-1 (red), persistent (dark red), and phase-2 (blue) P-element insertions in ORCs and shared sites. (**C**) Average proportion of lost P-element insertions out of all phase-1 P-element insertions by annotation feature, ORCs, and shared sites. (**D**) Replicate frequency spectrum (31) of persistent P-element insertions inside and outside of ORCs and shared sites (!ORC & !shared: P-element insertions that are located outside of OCRs and shared sites, ORC & !shared: P-elements insertions that are restricted to ORCs only, etc.). For separate analysis, please see Supplementary Figure S3.

With *f_l_* being the total genomic feature length (e.g. total length of all exons, Supplementary File 5), *P_obs-total_* the total number of observed P-element insertions on the main chromosome arms, and *G_l_* the total length of the main chromosome arms. The enrichment of P-element insertions (Figure 2A, B) is defined as the fraction of observed (*P_obs_*) to expected number of P-element insertions for the genomic feature of interest: $\frac{{{P}_{obs}}}{{{P}_{exp}}}$.

We used a Cochran–Mantel–Haenszel test (CMH-test) to test, whether the abundance of lost and persistent P-element counts grouped by annotation feature differ across experimental populations. The CMH-test allows to test of independence of stratified categorical data (i.e. P-element type abundances across replicate populations) (45).

##### Origin recognition complex (ORC) binding sites

For experimental populations, we detected ORCs in *D. simulans* scrutinizing ORC sequences extracted from the *D. melanogaster* reference genome (v5.53) (3) as described previously for the natural South African population (4). Following (4,46), we mapped these sequences to our *D. simulans* reference genome (33) using bwa bwasw (v0.7.17; *–M*) (36). We sorted and merged overlapping ORC sequences with bedtools merge (v2.29.2) (44) and retained only ORC regions with a minimum length of 100 bp, resulting in 5788 non-overlapping ORC regions for experimental populations (Supplementary File 6). We used the R-package GenomicRanges (v1.42.0) (47) to calculate the overlap in base pairs of ORCs with different annotation tracks in *D. simulans* (33). We note that this analysis assumes that ORCs are shared between *D. melanogaster* and *D. simulans*, but given the high sequence conservation between both species (48), we do not consider that this assumption affected our analysis. Rather, if *D. melanogaster* ORCs are no longer functional in *D. simulans*, this makes our analyses conservative, as non-functional ORCs are included into the ORC set.

For enrichment analyses (Figure 2B), we calculated the number of expected P-element insertions in ORCs for each replicate population using equation (1) with *f_l_* being the total genomic length of all ORCs. For phase-2 P-element insertions *f_l_* is reduced by the total length of ORCs that are already occupied by phase-1 P-element insertions.

##### Shared sites

We further tested whether P-element insertions are enriched in P-element insertion sites shared between *D. melanogaster* and *D. simulans* from South Africa (4) (‘shared sites’). For this, we extracted P-element insertions that have been previously classified as ‘shared sites’ (±1000 bp) from the *D. melanogaster* reference genome (v5.53) (4) using bedtools getfasta (v2.29.2) (44) and mapped them with bwa bwasw (v0.7.17 *-M*) (49) to our *D. simulans* reference genome (33). We used samtools (v1.11) (42) to filter for primary alignments with a minimum mapping quality of 15. We used bedtools (v2.29.2) (44) to sort and merge overlapping regions, resulting in 337 regions classified as shared sites for experimental populations (Supplementary File 7). We used the R-package GenomicRanges (v1.42.0) (47) to calculate the overlap in base pairs of shared sites with different annotation features of the *D. simulans* annotation (33).

We used Fisher's Exact Test to assess whether shared sites have a different frequency of persistent P-elements than 3′UTR, CDS, 5′UTR, introns and intergenic regions in experimental populations (Figure 2C). For each pairwise test (e.g. shared sites against CDS), we excluded P-elements in shared sites that are also positioned in the genomic feature of interest. We used the Benjamini-Hochberg correction to adjust p-values for multiple testing across all replicate populations.

#### Aggregation calculation

Insertion bias into ORCs/shared and selection operating against P-element insertions outside of ORCs/shared sites results in an aggregation of P-element insertions in ORC/shared sites. To disentangle insertion bias from selection, we contrasted the aggregation of P-elements into these regions between phase 1 and phase 2. If no selection is operating, the same level of aggregation is expected in phase 1 and phase 2, but with selection, the observed aggregation should be stronger in phase 2. We determined the aggregation of P-element insertions in a genomic region as the fraction of P-element insertions occurring into the respective region of interest (i.e. either ORCs or shared sites in our analysis). The calculation of the aggregation in phase 1 is rather straightforward: The aggregation is simply the number of P-element insertions into a genomic region divided by the total number of detected phase-1 P-element insertions. The inference of aggregation in phase 2 is a bit more complicated, because we need to account for the possibility that lost phase-1 P-element insertions are restored by newly acquired P-element insertions during phase 2. For this, we first calculated the fraction of phase-1 P-element insertions outside ORCs and shared sites that are rediscovered after phase 2 (i.e. the fraction of persistent P-elements). This quantity—which we termed the ‘rediscovery rate’—provides due to low re-insertion probabilities (caused by a lack of strong insertion bias) a good estimator for the expected fraction of persistent phase-1 P-elements in ORCs/shared sites if P-elements in these genomic regions would evolve under the same evolutionary forces (i.e. identical selection pressure and no re-insertions due to insertion bias). The difference between the actually observed and – based on the rediscovery rate – expected number of persistent P-element insertions in ORCs/shared sites in phase 2 provides an estimate for the re-insertion rate. This calculation assumes a constant selection pressure on P-element insertions regardless of their genomic position (i.e. inside/outside of ORCs/shared sites). As a next step, we estimated the number of P-element insertions that occurred during phase 2 as the sum of observed phase-2 P-element insertions and the number of expected re-insertions. Finally, the aggregation of P-elements into ORCs/shared sites during phase 2 is estimated as the fraction of P-insertions in these regions, corrected for re-insertions. We note that the aggregation analysis is not affected by non-uniform insertion bias among ORCs/shared sites.

### Diversity measures in experimental populations

**Figure 3.**
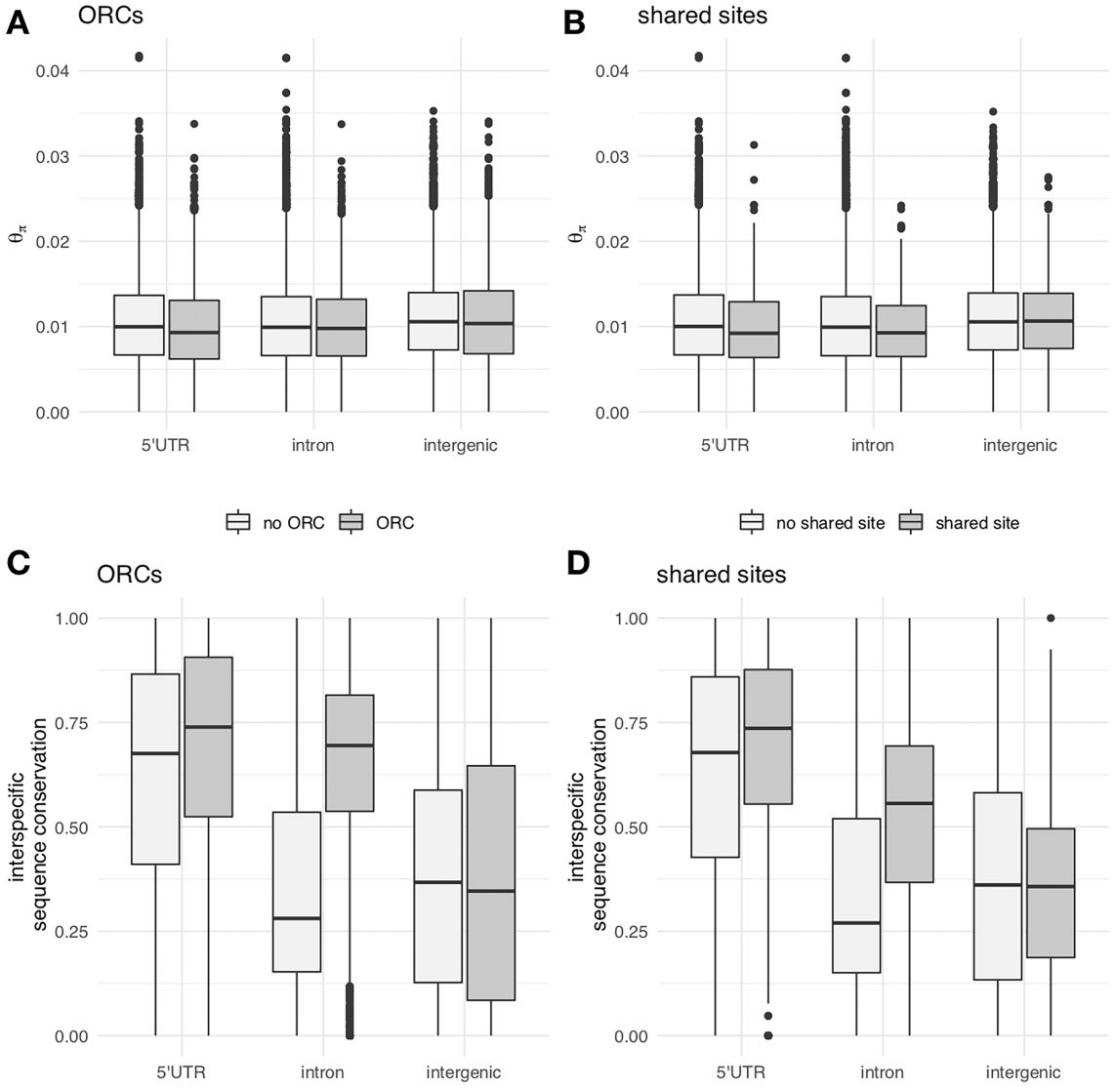
Intraspecific polymorphism (${{\mathrm{\theta }}}_{\mathrm{\pi }})$ and interspecific sequence conservation measurements. (**A**) Average θ_π_ estimates across ancestral experimental populations (*n* = 3) for three different annotation features (x-axis), grouped by an overlap with ORCs. We chose 5′UTR, introns, and intergenic regions for the comparison because more than 90% of the regions annotated as ORCs or shared sites overlap with these three annotation features. (**B**) Average θ_π_ estimates across ancestral experimental populations (*n* = 3) for three different annotation features (x-axis), grouped by an overlap with shared sites (**C**) Interspecific sequence conservation for three different annotation features, grouped by an overlap with ORCs. (**D**) Interspecific sequence conservation for three different annotation features, grouped by an overlap with shared sites

We evaluated whether base substitutions, similar to P-elements, experience less purifying selection in ORCs and shared sites. For this, we investigated the level of intraspecific polymorphisms in the ancestral experimental populations (${\theta }_\pi$) and the level of interspecific sequence conservation (50) in ORCs and shared sites for three genomic features (5′UTR, introns, and intergenic regions; Figure 3; Supplementary Files 8–9). We chose 5′UTR, introns, and intergenic regions for the comparison because more than 90% of the regions annotated as ORCs or shared sites overlap with these three annotation features.

#### Intraspecific polymorphisms

To estimate the level of intraspecific polymorphisms in the ancestral populations, we first trimmed the already demultiplexed data with ReadTools (v1.5.2; *–minReadLength 50 –mottQualityThreshold 20 –disable5pTrim*) (34) and mapped the reads to the *D. simulans* reference genome (33) using novoalign (v3.03.02; *-i 400 100 -F STDFQ -o SAM -r RANDOM*) (Novocraft, 2015: http://www.novocraft.com/products/novoalign) on a Hadoop cluster with Distmap version 2.7.5 (51). We removed duplicates with picard (http://broadinstitute.github.io/picard, v2.23.8; *MarkDuplicates*) and clipped overlapping read pairs with bamUtil clipOverlap (v1.0.14) (52). We used samtools (v1.11) (42) to filter for properly paired reads and a mapping quality of at least 20. After sequence alignment, we used PoPoolation (53) to calculate unbiased ${\theta }_\pi$ estimates from the Pool-Seq data of the ancestral experimental populations. For each replicate population, we built a pileup with samtools (v1.11) (42) masked for low complexity regions (<= 200 bp; RepeatMasker, http://www.repeatmasker.org, v4.0.7) and Y-translocations (43). We calculated ${\theta }_\pi$ in non-overlapping windows of 2 kb along the genome with the *Variance-sliding.pl* script from PoPoolation (*–pool-size 955 –min-count 2 –min-coverage 10 –max-coverage 500 –min-covered-fraction 0.75 –min-qual 20*) (53). We created bed files for the 5′UTR, intron, and intergenic region annotations from the *D. simulans* annotation (4,33) with bedtools sort, merge, and complement (v2.29.2) (44). We calculated average ${\theta }_\pi$ across replicate populations weighted by the overlap in base pairs for different annotation features (e.g. intergenic regions) with bedtools intersect (v2.29.2) (44).

#### Interspecific sequence conservation measurement

We downloaded publicly available genome annotations of *D. melanogaster* (v6) (54) as well as evolutionary conservation measurements (conserved elements predicted by phastCons (50)) (55) from the UCSC Genome Browser (http://genome.ucsc.edu) with the UCSC Table Browser application (56). To determine the ratio of ORCs and shared sites that are covered by conserved elements (interspecific sequence conservation), we mapped the according sequences against v6 of the *D. melanogaster* reference genome (54) with bwa bwasw (v0.7.17 *-M*) (49). We filtered for primary alignments with a minimum mapping quality of 20 (42), before regions were sorted and merged (44). We used bedtools intersect (v2.29.2) (44) to calculate the ratio of overlap between different annotation features (i.e. 5′UTR, introns, intergenic region) with conserved elements (50). To determine the positions of *D. simulans* phase-1 P-element insertion sites in the *D. melanogaster* reference genome (v6) (54), we extracted phase-1 P-element insertions (±150 bp) from our *D. simulans* reference genome (33) with bedtools getfasta (v2.29.2) (44) and mapped them with bwa bwasw (v07.7.17 *-M*) (49) against the *D. melanogaster* reference genome. We used samtools (v1.11) (42) to filter for primary alignments and a minimum mapping quality of 15. We sorted and merged overlapping regions with bedtools (v2.29.2) (44) and retained only regions with a minimum length of 100 bp. This resulted in 1012 non-overlapping regions in the *D. melanogaster* reference genome that contained at least one *D. simulans* phase-1 P-element. We used the R-package GenomicRanges (v1.42.0) (47) to assess the overlap between phase-1 P-elements, ORCs, shared sites, and annotation features of the *D. melanogaster* reference genome.

## RESULTS

### P-element proliferates in *D. simulans* isofemale lines

Isofemale lines were established from a natural *D. simulans* population, which is being invaded by the P-element (4). Lines with an active P-element are expected to accumulate additional P-element copies until the piRNA defense system is established (57). We confirmed this anticipated increase in P-element copy number by sequencing three isofemale lines with an active P-element (4) after 2 and 6 years of maintenance. We estimated P-element copy number per haplotype for each time point with DeviaTE (35). The P-element copy number increased in all three lines to at least 11 copies per haplotype (Supplementary Figure S1, Supplementary File 1), which is broadly consistent with the distribution of P-elements in natural and experimental populations at the end of a P-element invasion (4,30). The increase in copy number confirms that the P-element has further spread in isofemale lines carrying the transposon.

### Heterogeneous distribution of P-element insertions

We combined isofemale lines with and without P-elements to set up three large experimental populations with a population size of 1250 individuals each. We performed Pool-Seq for the founder populations and three replicate populations, which evolved for 10 generations in a hot environment fluctuating between 18 and 28°C. We used PopoolationTE2 (41) to identify P-element insertion sites from Pool-Seq data of these three replicate populations after phase 1 (founder population, generation 0) and phase 2 (generation 10). We detected 1040 phase-1 P-element insertions on the main chromosome arms: 812 on the autosomes and 228 on the X-chromosome. After phase 1, we found a pronounced underrepresentation of P-element insertions in coding regions. (joint analysis: ${\chi }^2 = 45.524,\ df = 1,P \,<\, 0.001$, see Material & Methods: *Genomic features*, Supplementary File 2–3, see Supplementary Table S1 for replicate-specific (i.e. ‘separate’) analysis). The 5′ UTR harbored more P-element insertions than expected under a random insertion process (joint analysis: ${\chi }^2 = 129.29,\ df = 1,P \,<\, 0.001$, Supplementary Table S1 (separate analysis)). Since this excess of P-element insertions in the 5′UTR coincides with a previously reported insertion bias (2), we also measured whether the P-element is overrepresented in origin recognition complex (ORC) binding sites—genomic regions that are considered to be P-element insertion hotspots (3). As expected from the previously reported insertion preference (3), we found a pronounced overrepresentation of P-elements in ORCs (joint analysis: ${\chi }^2 = 303.29,\ df = 1,P \,<\, 0.001$, Supplementary Table S1 (separate analysis)).

### Purifying selection shapes the genome-wide P-element distribution

Phase-1 P-element insertions occurred at small effective population sizes (*N*_e_) with low selection efficacy and were subjected to efficient selection due to larger *N*_e_ in phase 2. Thus, the comparison of P-element distributions in phase 1 and phase 2 shows the impact of purifying selection on P-element insertions. We explored the fate of P-element insertions from phase 1, which either persisted from phase 1 to phase 2 (‘persistent’ P-element insertions) or got lost in phase 2 (‘lost’ P-element insertions, Figure 1). The abundance of persistent and lost P-elements differed significantly across genomic features (joint analysis: Cochran-Mantel-Haenszel Test, ${M}^2 = 69.249,\ df = 4,P \,<\, 0.001,$Supplementary Table S1 (separate analysis)) demonstrating a non-random loss of P-elements. Our analysis does not distinguish between P-element loss caused by purifying selection, random genetic drift, or excision. While selection is operating locally (i.e. varies across genomic features), the excision rate and random genetic drift should be homogeneous across the genome and thus, different genomic features are affected similarly by these random processes. Furthermore, the heterogeneous distribution of lost and persistent P-element insertions cannot be explained by a systematic difference in P-element frequencies across genomic features in phase 1 (Kruskal–Wallis rank sum test ,$\ {\chi }^2 = 8.7561,\ df = 4,P = \ 0.06749$, Supplementary Figure S2A).

Given this strong signal for heterogeneous selection across the genome, we determined the enrichment (=observed/expected) of persistent P-element insertions for different genomic features (Figure 2A). The enrichment of persistent P-element insertions in experimental populations is remarkably similar to enrichment of P-element insertions in a natural *D. simulans* population with an established P-element invasion (4): with the exception of intergenic regions and 5′UTRs (Figure 2A), persistent P-element insertions displayed signs of purifying selection (i.e. enrichment < 1) across the genome. We observed the lowest average fraction of persistent P-elements in coding regions, reflecting the expected strong selection against TE insertions in coding sequences (7,15). These results suggest that the majority of P-element insertions are deleterious. Furthermore, the very efficient selection against P-element insertions implies that unlike most de novo mutations, P-element insertions (58,59) are most likely not recessive, as the low population frequency of each insertion after phase 1 makes selection against recessive P-element insertions in phase 2 less efficient.

The pattern for ORCs was rather unexpected. The enrichment of persistent P-element insertions in ORCs was about 85% higher than the enrichment of phase-1 P-element insertions (Figure 2B) and resembled the enrichment of P-element insertions in ORCs of a natural *D. simulans* population (4). This increased over-representation indicates weaker purifying selection in ORCs than in the remaining genome. Yet, the enrichment analysis assumes that no new P-element insertions occurred during phase 2, which could restore a phase-1 P-element insertion after it was lost by drift, excision, or selection during phase 2. Hence, a strong insertion bias may be misinterpreted as a signal of low purifying selection. Because only 5.2% of the ORCs contained P-element insertions at the beginning of phase 2, such ‘recurrent insertions’ do not occur at a sufficiently high rate to explain the increased enrichment. Nevertheless, if the strength of insertion bias differs among ORCs, re-insertions in the same site are more likely and this analysis is not sufficient to distinguish between purifying selection and insertion bias.

With ORCs, a preferred P-element insertion target (2,3), experiencing less purifying selection than other genomic regions, we also evaluated another category of preferred insertion targets: sites that share P-element insertions between natural *D. melanogaster* and *D. simulans* populations (‘shared sites’). Because the split between *D. melanogaster* and *D. simulans* predates the P-element invasion in both species (4,28,60), the presence of shared P-element insertion sites has been attributed to a strong insertion bias of the P-element (4). We observed the same pattern for shared sites as for ORCs – the enrichment of persistent P-elements in shared sites overall agrees with the enrichment in a natural *D. simulans* population. The enrichment of persisting P-element insertions was even stronger in shared sites than in ORCs (162%, Figure 2B). Moreover, all genomic features, even introns and intergenic regions, lost more phase-1 P-element insertions than shared sites (joint analysis: *P_adj_* < 0.001 for all comparisons after multiple testing correction, pairwise Fisher's exact tests between each annotation feature and shared sites per experimental population; Figure 2C; Supplementary Table S1 (separate analysis)). We interpret this pattern as evidence for reduced selection against P-element insertions in ORCs/shared sites.

Further evidence for low purifying selection in ORCs and shared sites comes from the probability to detect the same persistent P-element insertion in multiple replicate populations after phase 2. P-elements subjected to purifying selection are either segregating at a low frequency in evolved replicates or they have already been removed in a subset of the replicates. In both cases, we expect that most of these P-elements will be detected in a single replicate population. Phase-1 P-element insertions exposed to strong purifying selection will be removed in all replicates during phase 2 and are classified as lost. We compared the percentage of four classes of persistent P-element insertions (within/outside ORCs/shared sites) that could be detected in one, two, and all three replicate experimental populations. Consistent with weaker purifying selection in ORCs/shared sites we observed that these genomic regions shared more persistent P-element insertions across at least two replicates than the remaining genome (Figure 2D). The comparison of ORCs and shared sites suggests weaker purifying selection in shared sites than in ORCs (Figure 2B–D).

The enrichment of persisting P-elements in ORCs/shared sites provides two important insights. First, the strength of selection against P-element insertions is heterogeneous across the entire genome. Second, the insertion bias of the P-element is probably overestimated when relative P-element abundances of natural populations are used.

Since insertion bias and low levels of purifying selection provide the same pattern of enrichment in ORCs and shared sites, we were interested to disentangle these two processes. In absence of reduced purifying selection in OCRs/shared sites, the aggregation of P-element insertions in ORCs/shared sites should be similar in phase 1 (with weak purifying selection due to small *N*_e_) and phase 2 (with stronger purifying selection due to larger *N*_e_). We estimate the aggregation of the P-element in a genomic region (i.e. ORCs/shared sites) as the fraction of P-element insertions occurring into this region. While the calculation of the level of aggregation in phase 1 is straightforward, estimating this quantity in phase 2 is complicated by the difficulty to distinguish truly persistent P-element insertions from P-element insertions that were lost in phase 2, but were replaced by new P-element insertions later on. Assuming that ORCs and shared sites experience the same evolutionary forces (i.e. purifying selection, drift, stochastic sampling during Pool-Seq) as other genomic regions, we calculated the ‘rediscovery rate’, which is the probability to detect a phase-1 P-element after phase 2. In phase 2, the total number of new P-element insertions into OCRs/shared sites can be calculated by adding the expected number of re-insertions (a quantity that can be calculated based on the rediscovery rate, see M&M section *Aggregation calculations* for details) to the observed number of P-element insertions. Consistent with weaker purifying selection in ORCs/shared sites, the level of aggregation increased by 75% (joint analysis: shared sites, SEM = 29%, Supplementary Table S1 (separate analysis of each replicate)) and 43% (ORCs, SEM = 12%, Supplementary Table S1 (separate analysis of each replicate)) between phase 1 and phase 2. Assuming that the insertion preference of the P-element does not change between phase 1 and phase 2, the increase in the level of aggregation can be attributed to reduced purifying selection in ORCs and shared sites.

### Purifying selection operates differently on TE insertions and base substitutions

We evaluated whether base substitutions, similar to P-elements, experience less purifying selection in ORCs/shared sites. We found no evidence for relaxed purifying selection against base substitutions. Neither were intraspecific polymorphisms (${\theta }_\pi )$ elevated nor was interspecific sequence conservation (50) lower in ORCs and shared sites (Figure 3). This pattern persisted when we only considered ORCs and shared sites with persistent P-element insertions (Supplementary Figure S4 (joint analysis), Supplementary Figure S5 (separate analysis)). Interestingly, our findings suggest an opposite trend, indicating potentially enhanced purifying selection on base substitutions within ORCs/shared sites. It is important to acknowledge, however, that our study was not explicitly designed to investigate the underlying mechanisms driving these patterns. Overall, our result suggests that for many genomic regions selection against TE insertions is driven by evolutionary forces that differ from selection against base substitutions.

Ectopic recombination is frequently considered a major selection force against TE insertions (9–11). To protect TE insertions in ORCs and shared sites from ectopic recombination, a lower local recombination rate would be required. Because the recombination map in *D. simulans* is rather uniform across almost the entire chromosome arms (32,61) comparison of the P-element density in genomic regions with high and low recombination rates is not very powerful. Contrasting P-element insertions in regions with low recombination rates against remaining P-element insertions results in no difference between persistent and lost P-element insertions (joint analysis: Cochran–Mantel–Haenszel test, ${M}^2 = 1.2501,\ df = 1,P = 0.2635$, Supplementary Table S1 (separate analysis)). Furthermore, we cannot test the presence of recombination cold spots at ORCs and shared sites, because the currently available recombination map for *D. simulans* is too coarse (32).

## DISCUSSION

Contrasting the P-element insertions in populations with low and strong selection efficacy suggested that ORCs and shared sites are not only preferred insertion targets of the P-element, but P-element insertions in these genomic regions are more likely to be tolerated than in other parts of the genome.

One key assumption of our analyses is that properties of P-element insertions such as population frequencies, and insertion probabilities inside and outside of ORCs/shared sites were the same during phase 1. In fact, all P-element insertions occur only at a low frequency during phase 1 (Supplementary Figure S2). Nevertheless, P-element insertions in ORCs and shared sites have slightly higher frequencies than those outside (Supplementary Figure S2B). This higher frequency can be explained by the insertion bias, which results in independent P-element insertions at the same site in different isofemale lines. We caution, that a higher population frequency of P-element insertions in ORCs and shared sites results in a lower rate of loss of P-element insertions due to drift which may lead to a higher re-discovery rate, even without a difference in purifying selection. However, the accuracy of P-element insertion frequency estimates is constrained by the physical coverage of the data. Since low frequency estimates are particularly sensitive, we caution that potential differences in population frequency may not be very robust.

Nevertheless, the comparison of the enrichment in natural populations to our experiment suggests that the observed differences between ORCs/shared sites and the rest of the genome are not an artifact of the starting conditions in phase 1. The enrichment pattern for different genomic features is very similar between persistent P-elements in our laboratory experiment and P-elements in a natural *D. simulans* population with a fully established P-element invasion (Figure 2A). This suggest that our experiment nicely mirrors the selective forces operating on P-elements in natural populations. Importantly, we also observed a similar consistency between natural populations and our experiment for OCRs/shared sites (Figure 2B). If the small population frequency differences in phase 1 would have biased our analysis, the enrichment for ORCs/shared sites would have been more pronounced in our experiment than in natural populations. As this is not the case, we conclude that our enrichment estimates are robust to the subtle population frequency differences after phase 1. This implies that our conclusion that P-element insertions in ORCs/shared sites are subject to relaxed purifying selection remains unaffected.

The coincidence of genomic regions with relaxed purifying selection against P-element insertions and sites with previously reported insertion preferences is remarkable. We propose that the benefit of insertions into genomic regions with reduced selection efficacy provides a strong selection force that may have resulted in the evolution of insertion bias. Hence, insertion bias and reduced purifying selection should not be treated as two distinct phenomena, but rather be considered as cause and consequence. This hypothesis could be validated if distantly related P-elements have a different insertion bias than *D. melanogaster/D. simulans* P-elements. An experimental invasion of such a P-element in *D. melanogaster/D. simulans* would reveal the differences between insertion bias and purifying selection even more strongly than the experiments of this study.

One central unanswered question emerged from our study: Why do ORCs and shared sites experience less purifying selection than other genomic regions; and why does this only hold for P-element insertions and not for base substitutions? Since other TEs differ from the P-element in their transposition mechanisms, transposition rates, and genomic distributions, we hypothesize that selection pressures may be highly variable among TEs. We propose that heterogeneous selection may reflect the diversity of regulatory elements in different TEs (1,62). If these TE-encoded regulatory elements affect genes in their proximity, this will be typically deleterious and trigger purifying selection. In this case, we expect the weakest purifying selection pressure in genomic regions least responsive to regulatory elements introduced by specific TEs, which could also explain the heterogeneous distribution of different TEs in the genome. Whether selection really differs between TE families could be easily tested by introducing novel TEs into a host and following them in a similar experimental design as we introduced in this study. In addition to the spatially variable selection against P-elements in the genome, the observed discrepancy between base substitutions and P-element insertions may be affected by mutation rate heterogeneity (63).

Beyond TE dynamics, our study nicely demonstrates the challenge to distinguish between mutational bias and patterns of selection – especially when the inference is restricted to polymorphism and divergence data. We showed that at least for mutation processes occurring at sufficiently high rates, E&R provides a powerful experimental framework to scrutinize current views about the relative contribution of selection and mutational bias.

## Supplementary Material

gkad635_Supplemental_Files

## Data Availability

Information regarding data availability and processing steps of the isofemale lines can be found in Kofler *et al.* (4) and Howie *et al.* (32). Raw reads for the experimental populations are available from the European Nucleotide Archive under project accession number PRJEB54573. P-element insertion sites in the experimental populations, ORCs and shared sites annotations in *D. simulans* (BED format), intraspecific polymorphism estimates, and interspecific sequence conservation measurements are provided as Supplementary Files. Drosophila .svg graphics in the graphical abstract were obtained from https://doi.org/10.7875/togopic.2022.354 under the Creative Commons Attribution 4.0 International license. All scripts that are necessary to reproduce the results are available at SourceForge (https://sourceforge.net/projects/pelement-select/, last accessed: 5/5/2023).

## Notes

Present address: Anna M. Langmüller, Department of Computational Biology, Cornell University, Ithaca, 14850 NY, USA.
