# Supplementary material for: The genomic distribution of transposable elements is driven by spatially variable purifying selection
Source: Nucleic Acids Res. 2023 Aug 10;51(17):9203–13. doi: 10.1093/nar/gkad635 (PMC10516647; doi:10.1093/nar/gkad635)
Supplement: gkad635_Supplemental_Files [file gkad635_supplemental_files.zip › PFlH_AML_MS_v20-Supp.pdf]

Supplementary Figures

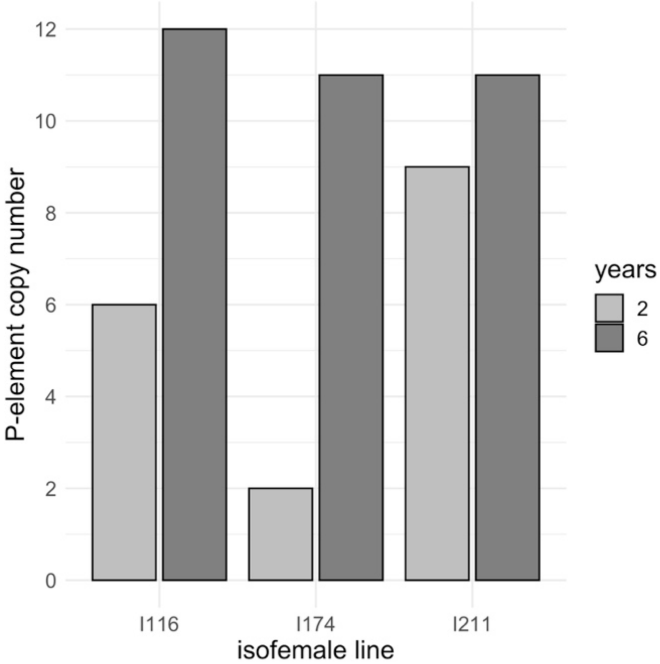

Figure S1. Estimated P-element copy number per haplotype in three isofemale lines after 2 (light grey) and 6 (dark grey) years at small population sizes (N ~ 40 – 50 individuals).

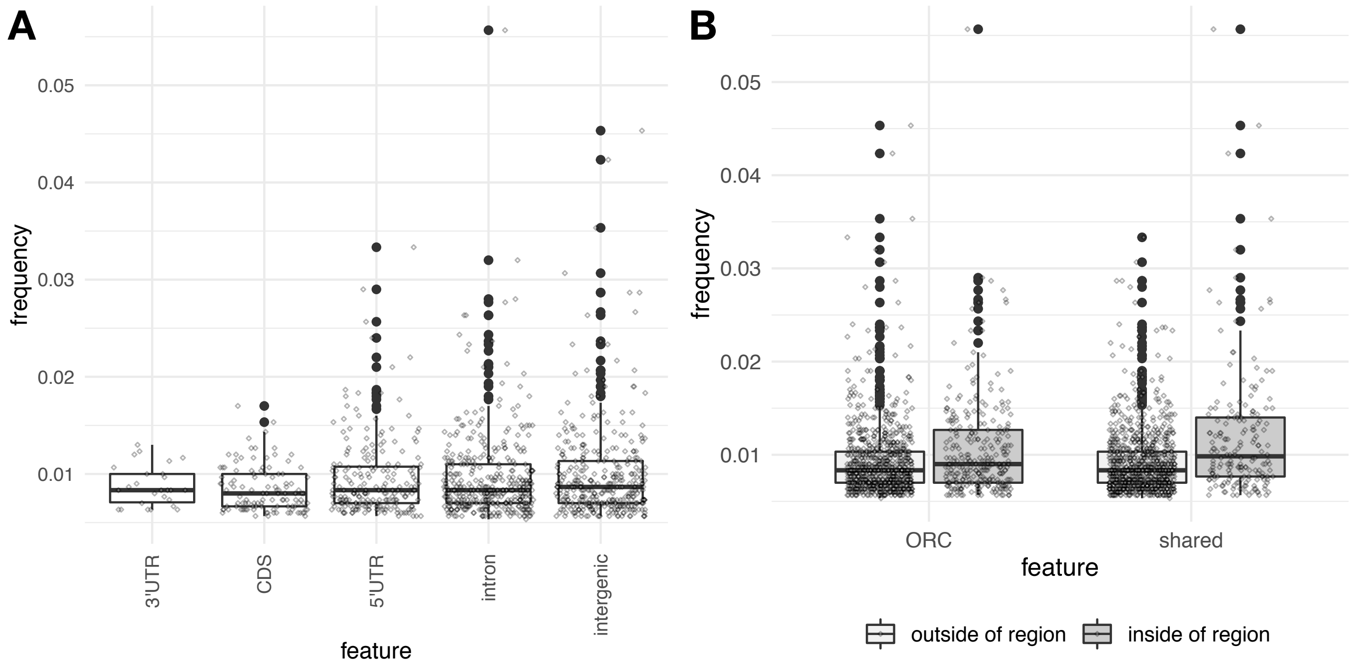

Figure S2. Phase 1 P-element population frequency estimates. (A) across genomic features (B) inside/outside of ORCs/shared sites. P-element insertions have slightly higher frequencies in ORCs (Wilcoxon rank sum test; *W=94247*; *P<0.001*) and shared sites (Wilcoxon rank sum test; *W=59026*; *P<0.001*)

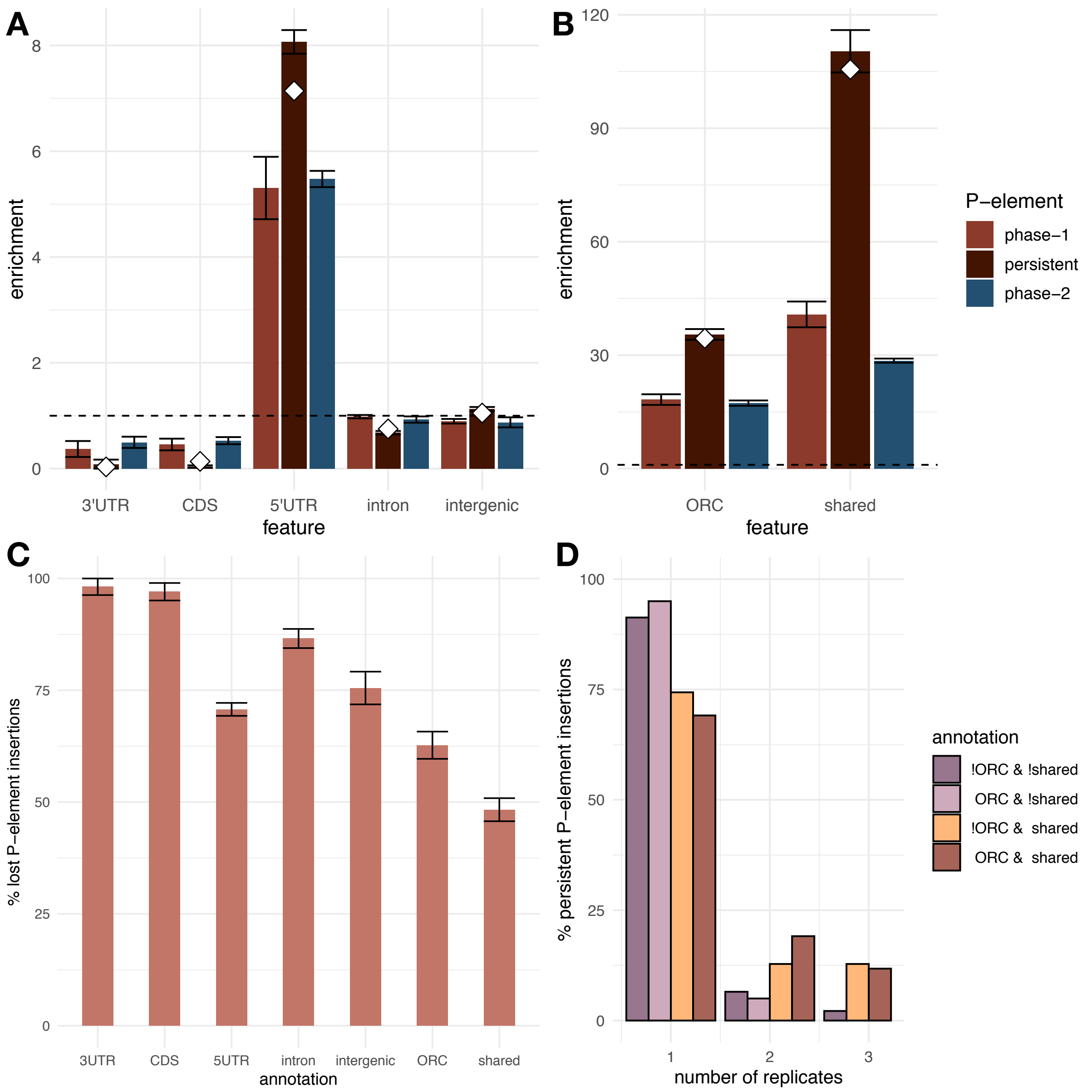

Figure S3. Characteristics of P-elements in phase 1 and phase 2: separate analysis. (A) Average enrichment (=observed/expected) of different P-element classes by annotation feature across three replicate populations. The horizontal dashed black line marks a homogeneous distribution (i.e., no insertion bias or selection, observed = expected). Nevertheless, since we consider the enrichment in the 5' UTR to reflect reduced purifying selection in combination with insertion bias, the lower enrichment values of all other functional classes indicate purifying selection. White diamonds display the enrichment of P-elements across genomic features for a natural *D. simulans* population from South Africa with an established P-element invasion (1). The error bars show the standard error of the mean across experimental populations. (B) Average enrichment of all phase-1 (red), persistent (dark red), and phase-2 (blue) P-element insertions in ORCs and shared sites. (C) Average proportion of lost P-element insertions out of all phase-1 P-element insertions by annotation feature, ORCs, and shared sites. (D) Replicate frequency spectrum of persistent P-element insertions inside and outside of ORCs and shared sites (!ORC & !shared: P-element insertions that are located outside of OCRs and shared sites, ORC & !shared: P-elements insertions that are restricted to ORCs only, etc.)

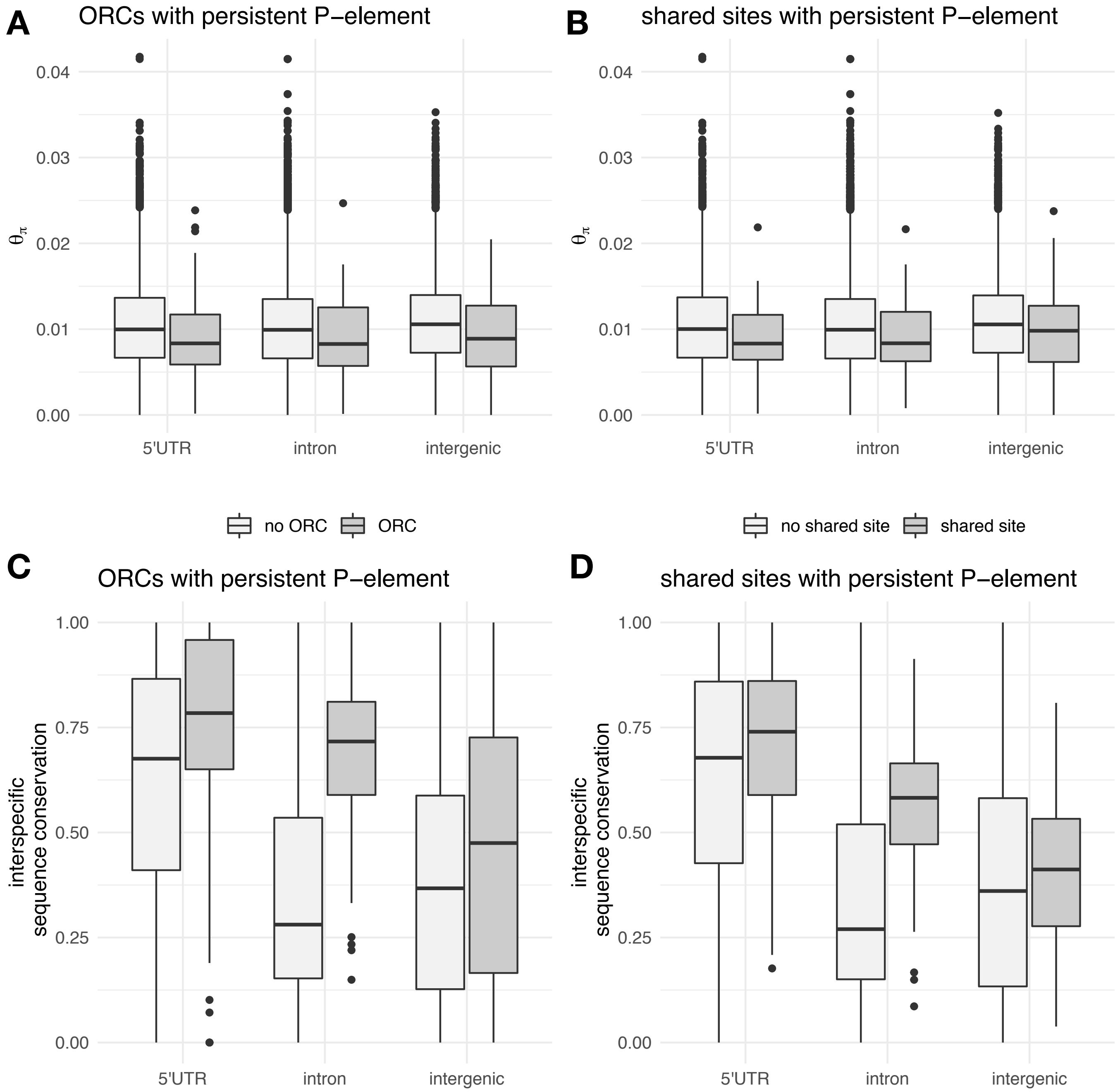

Figure S4. Intraspecific polymorphism (θ_π) and interspecific sequence conservation measurements: joint analysis. Only ORCs or shared sites with persistent P-element insertions are considered. (A) Average θ_π estimates across ancestral experimental populations (n=3) for three different annotation features (x-axis), grouped by an overlap with ORCs. We chose 5'UTR, introns, and intergenic regions for the comparison because more than 90% of the regions annotated as ORCs or shared sites overlap with these three annotation features. (B) Average θ_π estimates across ancestral experimental populations (n=3) for three different annotation features (x-axis), grouped by an overlap with shared sites. (C) Interspecific sequence conservation for three different annotation features, grouped by an overlap with ORCs. (D) Interspecific sequence conservation for three different annotation features, grouped by an overlap with shared sites.

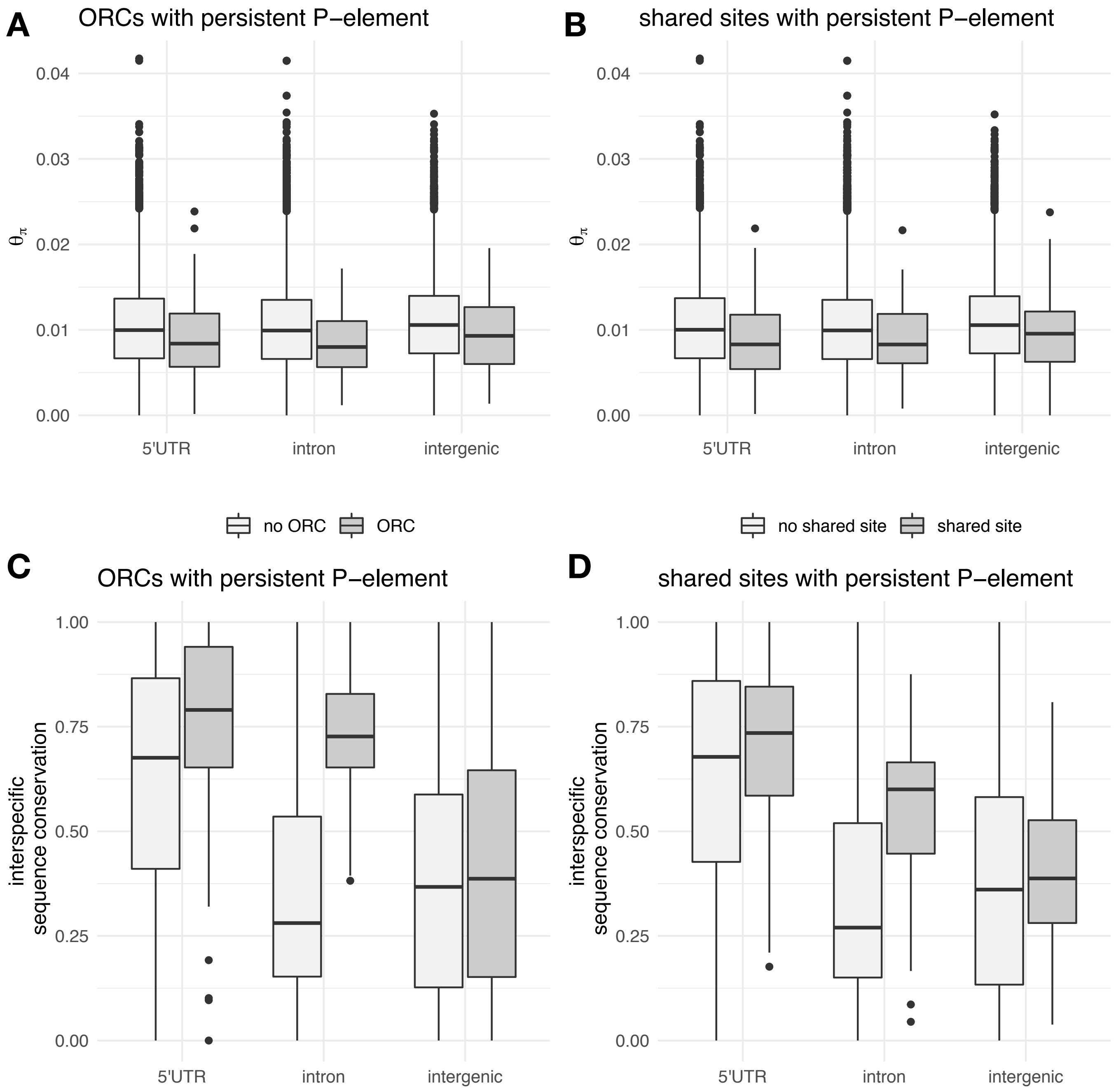

Figure S5. Intraspecific polymorphism (θ_π) and interspecific sequence conservation measurements: separate analysis. Only ORCs or shared sites with persistent P-element insertions are considered. (A) Average θ_π estimates across ancestral experimental populations (n=3) for three different annotation features (x-axis), grouped by an overlap with ORCs. We chose 5'UTR, introns, and intergenic regions for the comparison because more than 90% of the regions annotated as ORCs or shared sites overlap with these three annotation features. (B) Average θ_π estimates across ancestral experimental populations (n=3) for three different annotation features (x-axis), grouped by an overlap with shared sites. (C) Interspecific sequence conservation for three different annotation features, grouped by an overlap with ORCs. (D) Interspecific sequence conservation for three different annotation features, grouped by an overlap with shared sites.

Supplementary Tables

Table S1. Separate analysis overview.

Description (see main text for details)	Test Statistic	Degrees of Freedom	P-value	Remarks
Depletion of phase-1 P-element insertions in CDS per replicate (n=3)	$\chi^2 = 15.379$<br>$\chi^2 = 31.209$<br>$\chi^2 = 11.122$	df = 1<br>df = 1<br>df =1	$P_{adj}<0.001$<br>$P_{adj}<0.001$<br>$P_{adj}<0.001$	FDR corrected P-values (n=3)
Enrichment of phase-1 P-element insertions in 5'UTRs per replicate (n=3)	$\chi^2 = 58.152$<br>$\chi^2 = 56.349$<br>$\chi^2 = 48.814$	df =1<br>df =1<br>df =1	$P_{adj}<0.001$<br>$P_{adj}<0.001$<br>$P_{adj}<0.001$	FDR corrected P-values (n=3)
Enrichment of phase-1 P-element insertions in ORCs per replicate (n=3)	$\chi^2 = 126.45$<br>$\chi^2 = 113.54$<br>$\chi^2 = 138.74$	df =1<br>df =1<br>df =1	$P_{adj}<0.001$<br>$P_{adj}<0.001$<br>$P_{adj}<0.001$	FDR corrected P-values (n=3)
Abundance of lost/persistent P-element insertions across genomic features	$M^2 = 138.74$	df = 4	P<0.001	CMH test across replicates
Depletion of lost P-element insertions in shared sites	pairwise Fisher's Exact Tests between annotation feature and shared sites	df = 1	$P_{adj}<0.001$*	FDR corrected P-values (n=14)<br><br>* except for 3'UTR in one replicate population
Change in aggregation for ORCS	average increase: 19.9 % (SEM = 14.3 %)	--	--	--
Change in aggregation for shared sites	average increase: 29.5 % (SEM = 24.9 %)	--	--	--
Abundance of lost/persistent P-element insertions in regions of low recombination	$M^2 = 0.38792$	df = 1	P=0.5334	CMH test across replicates

Supplementary Files

Supplementary File 1. Estimated P-element copy number per haplotype for three isofemale lines after 2 and 6 years of maintenance. line_ID= isofemale line identifier; Pele_CN= estimated P-element copy number.

Supplementary File 2. P-element insertions on the main chromosome arms in phase 1 and phase 2 (joint analysis). id = P-element identifier; chr= chromosome; pos = genomic position (1-based); gen = generation (0 or 10); rep = replicate population (3, 4, or 5); type = P-element type ("seg" corresponds to "persistent", "ev-new" corresponds to "phase-2" in the main text); orc = boolean variable indicating whether ORCs overlap with the P-element insertion site; shared = boolean variable indicating whether shared sites overlap with the P-element insertion site; annotation = genomic annotation feature.

Supplementary File 3. P-element insertions on the main chromosome arms after phase 1 with replicate-specific population frequency estimates ("freq" column).

Supplementary File 4. P-element insertions on the main chromosome arms in phase 1 and phase 2 (separate analysis).

Supplementary File 5. Total length of different genomic annotation features on the main chromosome arms of *D. simulans*.

Supplementary File 6. ORCs annotation of experimental *D. simulans* populations (BED format).

Supplementary File 7. Shared sites annotation of experimental *D. simulans* populations (BED format).

Supplementary File 8. Average level of intraspecific polymorphisms across three ancestral experimental populations for three genomic features inside/outside of ORCs and shared sites (BED format). pi = average level of intraspecific polymorphism; annotation = genomic annotation feature; region = categorical variable indicating whether region overlaps with ORCs or shared sites.

Supplementary File 9. Interspecific sequence conservation in ORCs and shared sites for three genomic features (BED format). r = interspecific sequence conservation; annotation = genomic annotation feature; region = categorical variable indicating whether region overlaps with ORCs or shared sites.

Supplementary References

1. Kofler,R., Hill,T., Nolte,V., Betancourt,A.J. and Schlötterer,C. (2015) The recent invasion of natural *Drosophila simulans* populations by the P-element. *Proc. Natl. Acad. Sci.*, **112**, 6659 LP – 6663.
